# *N*′-(5-Bromo-2-hy­droxy­benzyl­idene)-4-nitro­benzohydrazide methanol monosolvate

**DOI:** 10.1107/S1600536811030108

**Published:** 2011-07-30

**Authors:** Wei-Hua Liu, Shuang-Ju Song, Jing-Jun Ma

**Affiliations:** a Hebei Key Laboratory of Bioinorganic Chemistry, College of Sciences, Agricultural University of Hebei, Baoding 071001, People’s Republic of China

## Abstract

In the title compound, C_14_H_10_BrN_3_O_4_·CH_4_O, the benzohydrazide mol­ecule is nearly planar [maximum deviation = 0.110 (2) Å]. The mean planes of the two benzene rings make a dihedral angle of 8.4 (3)°. In the benzohydrazide mol­ecule, there is an intra­molecular O—H⋯N hydrogen bond and the NH group is hydrogen bonded to the methanol solvent mol­ecule. In the crystal, inter­molecular O—H⋯O hydrogen bonds involving the methanol solvent mol­ecule link the benzohydrazide mol­ecules to form chains which propagate along the *a* axis.

## Related literature

For the biological activities of benzohydrazide compounds, see: El-Sayed *et al.* (2011 ▶); Horiuchi *et al.* (2009 ▶). For coordination compounds of benzohydrazide compounds, see: El-Dissouky *et al.* (2010 ▶); Zhang *et al.* (2010 ▶). For standard bond distances, see: Allen *et al.* (1987 ▶). For related structures, see: Suleiman Gwaram *et al.* (2010 ▶); Dai & Mao (2010 ▶); Ban (2010 ▶).
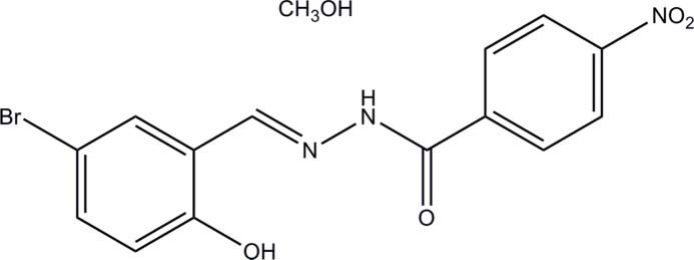


## Experimental

### 

#### Crystal data

C_14_H_10_BrN_3_O_4_·CH_4_O
*M* *_r_* = 396.20
Monoclinic, 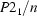

*a* = 6.660 (2) Å
*b* = 19.068 (3) Å
*c* = 12.730 (2) Å
β = 93.222 (2)°
*V* = 1614.1 (6) Å^3^
*Z* = 4
Mo *K*α radiation
μ = 2.58 mm^−1^
*T* = 298 K
0.17 × 0.13 × 0.12 mm

#### Data collection

Bruker SMART 1K CCD area-detector diffractometer
Absorption correction: multi-scan (*SADABS*; Sheldrick, 1996 ▶) *T*_min_ = 0.668, *T*_max_ = 0.747
8673 measured reflections
3442 independent reflections
1824 reflections with *I* > 2σ(*I*)
*R*_int_ = 0.062

#### Refinement

*R*[*F* ^2^ > 2σ(*F* ^2^)] = 0.049
*wR*(*F* ^2^) = 0.101
*S* = 0.95
3442 reflections
222 parameters
1 restraint
H atoms treated by a mixture of independent and constrained refinement
Δρ_max_ = 0.31 e Å^−3^
Δρ_min_ = −0.43 e Å^−3^

### 

Data collection: *SMART* (Bruker, 2007 ▶); cell refinement: *SAINT* (Bruker, 2007 ▶); data reduction: *SAINT*; program(s) used to solve structure: *SHELXS97* (Sheldrick, 2008 ▶); program(s) used to refine structure: *SHELXL97* (Sheldrick, 2008 ▶); molecular graphics: *SHELXTL* (Sheldrick, 2008 ▶); software used to prepare material for publication: *SHELXTL*.

## Supplementary Material

Crystal structure: contains datablock(s) I, global. DOI: 10.1107/S1600536811030108/su2299sup1.cif

Structure factors: contains datablock(s) I. DOI: 10.1107/S1600536811030108/su2299Isup2.hkl

Supplementary material file. DOI: 10.1107/S1600536811030108/su2299Isup3.cml

Additional supplementary materials:  crystallographic information; 3D view; checkCIF report

## Figures and Tables

**Table 1:** Hydrogen-bond geometry (Å, °)

*D*—H⋯*A*	*D*—H	H⋯*A*	*D*⋯*A*	*D*—H⋯*A*
N2—H2⋯O5	0.89 (1)	2.00 (2)	2.875 (4)	166 (4)
O1—H1⋯N1	0.82	2.03	2.737 (4)	143
O1—H1⋯O5^i^	0.82	2.51	2.952 (4)	115
O5—H5⋯O2^ii^	0.82	1.90	2.710 (4)	171

Symmetry codes: (i) 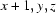; (ii) 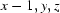.

**Fig. 1.**
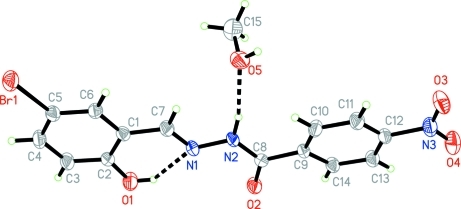
The molecular structure of the title compound, with the numbering scheme and displacement ellipsoids drawn at the 30% probability level. The N-H···O and O-H···N hydrogen bonds are shown as dashed lines.

**Fig. 2.**
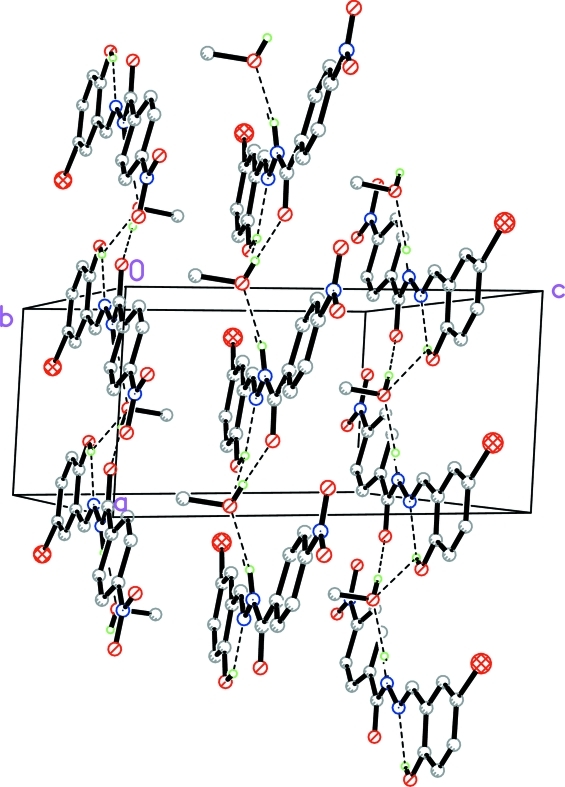
The crystal packing of the title compound, showing the N-H···O and O-H···O hydrogen-bonds (dashed lines) forming the chains propagating in [100]. H-atoms not involved in the hydrogen bonding have been omitted for clarity.

## supplementary crystallographic information

### Comment

Benzohydrazide compounds are well known for their biological activities (El-Sayed *et al.*, 2011; Horiuchi *et al.*, 2009). In addition, benzohydrazide compounds have also been used as versatile ligands in coordination chemistry (El-Dissouky *et al.*, 2010, Zhang *et al.*, 2010). As a contribution to a structural study on hydrazone compounds, we present here the crystal structure of the title compound, that was obtained as the product of the reaction of 5-bromosalicylaldehyde with 4-nitrobenzohydrazide in methanol.

The title compound contains a benzohydrazide molecule and a methanol solvent molecule of crystallization (Fig. 1). In the benzohydrazide molecule there is an intramolecular O-H···N hydrogen bond and NH group is hydrogen bonded to the methanol solvate molecule. The bond distances (Allen *et al.*, 1987) and angles are within normal ranges and agree well with the corresponding bond distances and angles reported in closely related compounds (Suleiman Gwaram *et al.*, 2010; Dai & Mao, 2010; Ban, 2010). The benzohydrazide molecule is nearly planar [maximum deviation of 0.110 (2) Å], with the mean planes of the two benzene rings making a dihedral angle of 8.4 (3)°.

In the crystal, intermolecular O—H···O hydrogen bonds involving the methanol solvate molecule link the benzohydrazide molecules to form chains which propagate along along the *a* axis direction (Table 1, Fig. 2).

### Experimental

To a methanol solution (20 ml) of 5-bromosalicylaldehyde (0.1 mmol, 20.1 mg) and 4-nitrobenzohydrazide (0.1 mmol, 18.1 mg), a few drops of acetic acid were added. The mixture was refluxed for 1 h and then cooled to room temperature. The white crystalline solid was collected by filtration, washed with cold methanol and dried in air. Single crystals, suitable for X-ray diffraction, were obtained by slow evaporation of a methanol solution of the product in air.

### Refinement

The NH H-atom was located in a difference Fourier map and was refined with a distance restraint, N-H = 0.90 (1) Å, and *U*_iso_(H) = 0.08 Å^2^. The OH and C-bound H atoms were positioned geometrically and refined using a riding model: O-H = 0.82 Å, C—H = 0.93 and 0.96 Å, for CH and CH_3_ H-atoms, respectively, with *U*_iso_(H) = k × *U*_eq_(O,C) where k = 1.5 for OH and CH_3_ H-atoms and k = 1.2 for all other H-atoms.

### Crystal data

**Table:**

C_14_H_10_BrN_3_O_4_·CH_4_O	*F*(000) = 800
*M**_r_* = 396.20	*D*_x_ = 1.630 Mg m^−^^3^
Monoclinic, *P*2_1_/*n*	Mo *K*α radiation, λ = 0.71073 Å
*a* = 6.660 (2) Å	Cell parameters from 1199 reflections
*b* = 19.068 (3) Å	θ = 2.6–24.7°
*c* = 12.730 (2) Å	µ = 2.58 mm^−^^1^
β = 93.222 (2)°	*T* = 298 K
*V* = 1614.1 (6) Å^3^	Block, yellow
*Z* = 4	0.17 × 0.13 × 0.12 mm

### Data collection

**Table:**

Bruker SMART 1K CCD area-detector diffractometer	3442 independent reflections
Radiation source: fine-focus sealed tube	1824 reflections with *I* > 2σ(*I*)
graphite	*R*_int_ = 0.062
ω scans	θ_max_ = 27.0°, θ_min_ = 2.1°
Absorption correction: multi-scan (*SADABS*; Sheldrick, 1996)	*h* = −8→8
*T*_min_ = 0.668, *T*_max_ = 0.747	*k* = −22→24
8673 measured reflections	*l* = −10→16

### Refinement

**Table:**

Refinement on *F*^2^	Primary atom site location: structure-invariant direct methods
Least-squares matrix: full	Secondary atom site location: difference Fourier map
*R*[*F*^2^ > 2σ(*F*^2^)] = 0.049	Hydrogen site location: inferred from neighbouring sites
*wR*(*F*^2^) = 0.101	H atoms treated by a mixture of independent and constrained refinement
*S* = 0.95	*w* = 1/[σ^2^(*F*_o_^2^) + (0.0375*P*)^2^] where *P* = (*F*_o_^2^ + 2*F*_c_^2^)/3
3442 reflections	(Δ/σ)_max_ = 0.001
222 parameters	Δρ_max_ = 0.31 e Å^−^^3^
1 restraint	Δρ_min_ = −0.43 e Å^−^^3^

### Special details

**Table:**

Geometry. All e.s.d.'s (except the e.s.d. in the dihedral angle between two l.s. planes) are estimated using the full covariance matrix. The cell e.s.d.'s are taken into account individually in the estimation of e.s.d.'s in distances, angles and torsion angles; correlations between e.s.d.'s in cell parameters are only used when they are defined by crystal symmetry. An approximate (isotropic) treatment of cell e.s.d.'s is used for estimating e.s.d.'s involving l.s. planes.
Refinement. Refinement of *F*^2^ against ALL reflections. The weighted *R*-factor *wR* and goodness of fit *S* are based on *F*^2^, conventional *R*-factors *R* are based on *F*, with *F* set to zero for negative *F*^2^. The threshold expression of *F*^2^ > σ(*F*^2^) is used only for calculating *R*-factors(gt) *etc*. and is not relevant to the choice of reflections for refinement. *R*-factors based on *F*^2^ are statistically about twice as large as those based on *F*, and *R*- factors based on ALL data will be even larger.

### Fractional atomic coordinates and isotropic or equivalent isotropic displacement parameters (Å^2^)

**Table:**

	*x*	*y*	*z*	*U*_iso_*/*U*_eq_	
Br1	0.68607 (7)	0.04160 (2)	0.91805 (4)	0.06305 (19)	
N1	0.9844 (5)	0.36314 (16)	0.8690 (2)	0.0446 (9)	
N2	0.8848 (5)	0.42751 (16)	0.8642 (3)	0.0451 (9)	
H2	0.7512 (18)	0.427 (2)	0.853 (3)	0.080*	
N3	0.5371 (7)	0.74331 (19)	0.8890 (3)	0.0577 (10)	
O1	1.2966 (4)	0.26998 (14)	0.8658 (3)	0.0627 (9)	
H1	1.2483	0.3094	0.8694	0.094*	
O2	1.1708 (4)	0.49115 (14)	0.8668 (2)	0.0563 (8)	
O3	0.3577 (5)	0.73870 (16)	0.9023 (3)	0.0786 (10)	
O4	0.6261 (5)	0.79787 (17)	0.8773 (3)	0.0821 (11)	
O5	0.4668 (4)	0.40586 (15)	0.8042 (3)	0.0661 (9)	
H5	0.3706	0.4278	0.8249	0.099*	
C1	0.9471 (6)	0.23839 (19)	0.8828 (3)	0.0391 (10)	
C2	1.1502 (6)	0.2216 (2)	0.8756 (3)	0.0433 (10)	
C3	1.2093 (6)	0.1525 (2)	0.8787 (3)	0.0572 (12)	
H3	1.3443	0.1414	0.8733	0.069*	
C4	1.0715 (7)	0.0993 (2)	0.8896 (3)	0.0581 (12)	
H4	1.1133	0.0528	0.8907	0.070*	
C5	0.8718 (6)	0.1156 (2)	0.8989 (3)	0.0448 (10)	
C6	0.8105 (6)	0.18444 (19)	0.8959 (3)	0.0424 (10)	
H6	0.6755	0.1951	0.9026	0.051*	
C7	0.8712 (6)	0.3099 (2)	0.8769 (3)	0.0450 (10)	
H7	0.7334	0.3171	0.8791	0.054*	
C8	0.9875 (6)	0.4882 (2)	0.8666 (3)	0.0411 (10)	
C9	0.8637 (5)	0.55344 (18)	0.8725 (3)	0.0384 (9)	
C10	0.6648 (6)	0.55357 (19)	0.8983 (3)	0.0483 (11)	
H10	0.6010	0.5113	0.9112	0.058*	
C11	0.5605 (6)	0.6152 (2)	0.9052 (3)	0.0518 (11)	
H11	0.4274	0.6148	0.9239	0.062*	
C12	0.6532 (6)	0.6769 (2)	0.8845 (3)	0.0445 (10)	
C13	0.8513 (6)	0.6794 (2)	0.8602 (3)	0.0504 (11)	
H13	0.9144	0.7221	0.8488	0.060*	
C14	0.9540 (6)	0.61711 (19)	0.8530 (3)	0.0468 (11)	
H14	1.0871	0.6178	0.8346	0.056*	
C15	0.4336 (8)	0.3910 (2)	0.6962 (4)	0.0810 (16)	
H15A	0.5205	0.4196	0.6565	0.122*	
H15B	0.2960	0.4008	0.6747	0.122*	
H15C	0.4618	0.3424	0.6838	0.122*	

### Atomic displacement parameters (Å^2^)

**Table:**

	*U*^11^	*U*^22^	*U*^33^	*U*^12^	*U*^13^	*U*^23^
Br1	0.0601 (3)	0.0462 (3)	0.0829 (4)	−0.0063 (2)	0.0049 (2)	0.0158 (3)
N1	0.042 (2)	0.0374 (19)	0.054 (2)	0.0118 (16)	−0.0029 (17)	0.0009 (16)
N2	0.0325 (18)	0.0361 (18)	0.066 (3)	0.0090 (17)	−0.0054 (18)	−0.0002 (17)
N3	0.074 (3)	0.045 (2)	0.054 (3)	0.017 (2)	0.002 (2)	0.0011 (18)
O1	0.0411 (17)	0.0452 (16)	0.102 (3)	−0.0002 (14)	0.0036 (17)	0.0050 (18)
O2	0.0337 (17)	0.0477 (16)	0.087 (2)	0.0049 (13)	0.0019 (15)	−0.0032 (15)
O3	0.066 (2)	0.071 (2)	0.100 (3)	0.0291 (19)	0.012 (2)	−0.0011 (19)
O4	0.103 (3)	0.0429 (19)	0.101 (3)	0.0126 (19)	0.007 (2)	0.0085 (18)
O5	0.0413 (18)	0.064 (2)	0.092 (3)	0.0117 (15)	−0.0014 (17)	−0.0201 (18)
C1	0.040 (2)	0.040 (2)	0.037 (3)	0.0072 (18)	0.0015 (19)	−0.0007 (18)
C2	0.040 (2)	0.039 (2)	0.051 (3)	0.0014 (19)	−0.005 (2)	0.0023 (19)
C3	0.038 (2)	0.042 (2)	0.091 (4)	0.010 (2)	−0.002 (2)	0.002 (2)
C4	0.057 (3)	0.037 (2)	0.080 (4)	0.007 (2)	0.001 (3)	0.007 (2)
C5	0.043 (3)	0.041 (2)	0.050 (3)	−0.0022 (19)	0.001 (2)	0.009 (2)
C6	0.038 (2)	0.039 (2)	0.051 (3)	−0.0006 (18)	0.002 (2)	0.001 (2)
C7	0.040 (2)	0.042 (2)	0.053 (3)	0.006 (2)	0.004 (2)	−0.002 (2)
C8	0.040 (3)	0.039 (2)	0.043 (3)	0.003 (2)	−0.005 (2)	−0.0016 (19)
C9	0.037 (2)	0.035 (2)	0.044 (3)	0.0050 (17)	0.0002 (19)	0.0004 (18)
C10	0.046 (3)	0.036 (2)	0.064 (3)	−0.0013 (19)	0.007 (2)	−0.006 (2)
C11	0.038 (2)	0.048 (3)	0.070 (3)	0.006 (2)	0.009 (2)	−0.008 (2)
C12	0.054 (3)	0.039 (2)	0.040 (3)	0.012 (2)	−0.004 (2)	−0.0030 (19)
C13	0.052 (3)	0.037 (2)	0.062 (3)	0.002 (2)	0.002 (2)	0.007 (2)
C14	0.037 (2)	0.039 (2)	0.064 (3)	0.0001 (19)	0.002 (2)	0.005 (2)
C15	0.090 (4)	0.070 (3)	0.083 (5)	−0.004 (3)	0.006 (3)	0.001 (3)

### Geometric parameters (Å, °)

**Table:**

Br1—C5	1.901 (4)	C4—C5	1.377 (5)
N1—C7	1.271 (4)	C4—H4	0.9300
N1—N2	1.395 (4)	C5—C6	1.375 (5)
N2—C8	1.343 (5)	C6—H6	0.9300
N2—H2	0.894 (10)	C7—H7	0.9300
N3—O4	1.211 (4)	C8—C9	1.497 (5)
N3—O3	1.219 (4)	C9—C10	1.383 (5)
N3—C12	1.486 (5)	C9—C14	1.384 (5)
O1—C2	1.353 (4)	C10—C11	1.370 (5)
O1—H1	0.8200	C10—H10	0.9300
O2—C8	1.222 (4)	C11—C12	1.362 (5)
O5—C15	1.409 (5)	C11—H11	0.9300
O5—H5	0.8200	C12—C13	1.373 (5)
C1—C6	1.390 (5)	C13—C14	1.377 (5)
C1—C2	1.398 (5)	C13—H13	0.9300
C1—C7	1.456 (5)	C14—H14	0.9300
C2—C3	1.375 (5)	C15—H15A	0.9600
C3—C4	1.379 (5)	C15—H15B	0.9600
C3—H3	0.9300	C15—H15C	0.9600
			
C7—N1—N2	115.0 (3)	C1—C7—H7	118.4
C8—N2—N1	121.1 (3)	O2—C8—N2	123.1 (4)
C8—N2—H2	121 (3)	O2—C8—C9	121.0 (4)
N1—N2—H2	117 (3)	N2—C8—C9	115.9 (3)
O4—N3—O3	124.7 (4)	C10—C9—C14	118.2 (3)
O4—N3—C12	117.9 (4)	C10—C9—C8	123.4 (3)
O3—N3—C12	117.4 (4)	C14—C9—C8	118.3 (3)
C2—O1—H1	109.5	C11—C10—C9	120.9 (4)
C15—O5—H5	109.5	C11—C10—H10	119.6
C6—C1—C2	118.7 (3)	C9—C10—H10	119.6
C6—C1—C7	118.2 (3)	C12—C11—C10	119.5 (4)
C2—C1—C7	123.1 (4)	C12—C11—H11	120.3
O1—C2—C3	116.7 (3)	C10—C11—H11	120.3
O1—C2—C1	123.7 (3)	C11—C12—C13	121.7 (4)
C3—C2—C1	119.6 (4)	C11—C12—N3	119.1 (4)
C2—C3—C4	121.1 (4)	C13—C12—N3	119.2 (4)
C2—C3—H3	119.5	C12—C13—C14	118.2 (4)
C4—C3—H3	119.5	C12—C13—H13	120.9
C5—C4—C3	119.6 (4)	C14—C13—H13	120.9
C5—C4—H4	120.2	C13—C14—C9	121.5 (4)
C3—C4—H4	120.2	C13—C14—H14	119.2
C6—C5—C4	120.0 (4)	C9—C14—H14	119.2
C6—C5—Br1	121.2 (3)	O5—C15—H15A	109.5
C4—C5—Br1	118.9 (3)	O5—C15—H15B	109.5
C5—C6—C1	121.0 (4)	H15A—C15—H15B	109.5
C5—C6—H6	119.5	O5—C15—H15C	109.5
C1—C6—H6	119.5	H15A—C15—H15C	109.5
N1—C7—C1	123.1 (4)	H15B—C15—H15C	109.5
N1—C7—H7	118.4		

### Hydrogen-bond geometry (Å, °)

**Table:**

*D*—H···*A*	*D*—H	H···*A*	*D*···*A*	*D*—H···*A*
N2—H2···O5	0.89 (1)	2.00 (2)	2.875 (4)	166 (4)
O1—H1···N1	0.82	2.03	2.737 (4)	143
O1—H1···O5^i^	0.82	2.51	2.952 (4)	115
O5—H5···O2^ii^	0.82	1.90	2.710 (4)	171

Symmetry codes: (i) *x*+1, *y*, *z*; (ii) *x*−1, *y*, *z*.
